# Intercellular Ca^2+^ signalling in the adult mouse cochlea

**DOI:** 10.1113/JP276400

**Published:** 2018-11-22

**Authors:** Piotr Sirko, Jonathan E Gale, Jonathan F Ashmore

**Affiliations:** ^1^ UCL Ear Institute 332 Gray's Inn Road London WC1X 8EE UK; ^2^ Department of Neuroscience Physiology & Pharmacology UCL, Gower St. London WC1E 6BT UK; ^3^ Department of Cell & Developmental Biology UCL, Gower St. London WC1E 6BT UK

**Keywords:** Cochlea, hearing, calcium waves, supporting cells

## Abstract

**Key points:**

- Intercellular Ca^2+^ waves are increases in cytoplasmic Ca^2+^ levels that propagate between cells.
- Periodic Ca^2+^ waves have been linked to gene regulation and are thought to play a crucial role in the development of our hearing epithelium, the organ of Corti and the acquisition of hearing.
- We observed regular periodic intercellular Ca^2+^ waves in supporting cells of an *ex vivo* preparation of the adult mouse organ of Corti, and these waves were found to propagate independently of extracellular ATP and were inhibited by the gap junction blockers 1‐octanol and carbenoxolone.
- Our results establish that the existence of periodic Ca^2+^ waves in the organ of Corti is not restricted to the prehearing period.

**Abstract:**

We have investigated wave‐like cytoplasmic calcium (Ca^2+^) signalling in an *ex vivo* preparation of the adult mouse organ of Corti. Two types of intercellular Ca^2+^ waves that differ in propagation distance and speed were observed. One type was observed to travel up to 100 μm with an average velocity of 7 μm/s. Such waves were initiated by local tissue damage in the outer hair cell region. The propagation distance was decreased when the purinergic receptor antagonists pyridoxalphosphate‐6‐azophenyl‐2′,4′‐disulfonic acid (PPADS; 50 μm) or suramin (150 μm) were added to the extracellular buffer. Immunocytochemical analysis and experiments with calcium indicator dyes showed that both P2X and P2Y receptors were present in supporting cells. A second class of waves identified to travel longitudinally along the organ of Corti propagated at a lower velocity of 1–3 μm/s. These ‘slow’ Ca^2+^ waves were particularly evident in the inner sulcus and Deiters’ cells. They travelled for distances of up to 500 μm. The slow Ca^2+^ signalling varied periodically (approximately one wave every 10 min) and was maintained for more than 3 h. The slow waves were not affected by apyrase, or by the P2 receptor agonists suramin (150 μm) or PPADS (50 μm) but were blocked by the connexin channel blockers octanol (1 mm) and carbenoxolone (100 μm). It is proposed that the observed Ca^2+^ waves might be a physiological response to a change in extracellular environment and may be involved in critical gene regulation activities in the supporting cells of the cochlea.

## Key points

- Intercellular Ca^2+^ waves are increases in cytoplasmic Ca^2+^ levels that propagate between cells.
- Periodic Ca^2+^ waves have been linked to gene regulation and are thought to play a crucial role in the development of our hearing epithelium, the organ of Corti and the acquisition of hearing.
- We observed regular periodic intercellular Ca^2+^ waves in supporting cells of an *ex vivo* preparation of the adult mouse organ of Corti, and these waves were found to propagate independently of extracellular ATP and were inhibited by the gap junction blockers 1‐octanol and carbenoxolone.
- Our results establish that the existence of periodic Ca^2+^ waves in the organ of Corti is not restricted to the prehearing period.

## Introduction

Increases in cytoplasmic intracellular calcium (Ca^2+^) concentrations which propagate from cell to cell are known as intercellular Ca^2+^ waves. They have been shown to be present in a variety of cell types including glia, vascular endothelial and smooth muscle cells, hepatocytes *in vivo*, pigment and Müller cells of the retina as well as in immature cochlear supporting cells (reviewed by Leybaert & Sanderson, 2012). Such waves are thought to be involved in the synchronization of cells across long distances and typically propagate either via gap junctions or via extracellular messenger release through unpaired gap junctions, or ‘hemichannels’. Such Ca^2+^ waves have also been shown to be triggered by cell damage (Gale *et al*. 2004; Anselmi *et al*. 2008).

One of the mechanisms of such wave propagation involves purinergic P2 receptors (Anselmi *et al*. 2008; Ceriani *et al*. 2016). It has been proposed that an initial local increase of extracellular ATP activates purinergic receptors, leading to an increase in free cytoplasmic Ca^2+^ concentration ([Ca^2+^]_i_). The increased [Ca^2+^]_i_ in turn triggers a release of the intracellular messengers inositol tris‐phosphate (IP_3_) into the cytoplasm and a release of ATP into the extracellular environment. [Ca^2+^]_i_ levels are then increased in neighbouring cells via at least two distinct mechanisms: (1) IP_3_ diffusion into neighbouring cells via gap junctions triggering an increase in [Ca^2+^]_i_ levels; and (2) ATP release into the extracellular environment activating P2 receptors on neighbouring cells and leading to a repeat of the cycle. The kinetics and propagation of the wave thus depend on multiple steps.

In the inner ear, spontaneous extracellular ATP‐mediated Ca^2+^ waves have been observed during development (Tritsch *et al*. 2007). The waves are observed before the onset of hearing in a transient structure (Kölliker's organ) present during development close to the row of inner hair cells (IHCs). Waves are present in those cells which express P2 receptors and which are located both medially and laterally from the IHCs. ATP is thought to be released spontaneously from cells to trigger Ca^2+^ waves. Waves are accompanied by cell shrinkage, which has been argued to be caused by osmolarity changes induced by the activation of Ca^2+^‐dependent TMEM‐16A Cl^−^ channels (Tritsch & Bergles, 2010; Wang *et al*. 2015). The resulting Cl^−^ efflux triggers a K^+^ efflux. The increase in K^+^ concentrations near the hair cell depolarizes it and triggers activity in the auditory system, contributing to the functional maturation of the system.

These Ca^2+^ waves increase in the first postnatal week and then subside around the time of hearing onset (Tritsch & Bergles, 2010). During this period Kölliker's organ cells are reported to undergo apoptosis (Kamiya *et al*. 2001) and are replaced by the inner sulcus (IS) found in mature hearing mammals.

In the region located lateral to the IHCs, known as the lesser epithelial ridge (LER), Ca^2+^ waves can be induced by damage or release of caged IP_3_ (Gale *et al*. 2004; Anselmi *et al*. 2008). Similarly to waves in the Kölliker's organ these waves also propagate, at least partially through ATP release into the extracellular environment and subsequent P2 receptor activation (Anselmi *et al*. 2008). We have suggested that these Ca^2+^ waves may be important for hair cell survival, and have shown that in the neonatal cochlea, the Ca^2+^ waves induce phosphorylation of cell survival factors such as JNK and ERK1/2, thus changing their molecular affinity and affecting cell survival pathways (Gale *et al*. 2004; Lahne & Gale, 2008).

To date, no spontaneous Ca^2+^ waves have been observed in the mature organ of Corti, although cochlear supporting cells are extensively coupled via gap junctions and express purinergic P2Y receptors involved in Ca^2+^ wave propagation in immature tissue (Lagostena *et al*. 2001; Jagger & Forge, 2006; Huang *et al*. 2010). Waves resembling those seen in the immature cochlea have been reported in explants of the adult gerbil cochlea when exposed to high levels of sound (Chan & Rouse, 2016). We show here, using a novel dissection technique which produces minimal disruption before cellular imaging, that a different class of wave, which we term ‘slow’ Ca^2+^ waves, can be observed in the adult mouse organ of Corti. We find that cytoplasmic Ca^2+^ levels increase following stimulation with extracellular ATP and that both P2X and P2Y receptors are present. By blocking the ATP receptor pathway, however, we show that such slower Ca^2+^ waves do not rely on extracellular ATP for propagation.

## Methods

### Ethical approval

All work was carried out under the UK Animals (Scientific Procedures) Act 1986 and approved by the UCL Biological Services Animal Ethics Committee.

### Dissection and tissue preparation

Wildtype [postnatal day (P)15–90] c57BL/6NCrL mice of either sex with normal hearing were killed by cervical dislocation in accordance with UK Home Office regulations. The hearing function of the c57BL/6NCrL mice was not tested systematically, but we note that the wild‐type mice used all responded to a simple acoustic startle sound. Most animals were in the age range P25–P35. The temporal bone containing the semicircular canals and the cochlea was removed and placed in extracellular solution containing (mm): 140 NaCl, 1.3 CaCl_2_, 1 mgCl_2_, 5.4 KCl, Hepes 10. The osmolarity of the extracellular solution was adjusted to 320 mosmol/l with glucose and the pH was adjusted to 7.4 with NaOH. The bone covering the apical turn of the organ of Corti and Reissner's membrane was removed using fine forceps to gain access to the scala media, exposing a small opening to observe the structures below between the 10% and 30% positions along the partition from the apex. In some experiments the tectorial membrane (TM) covering the organ of Corti was gently removed using a glass micropipette while observing the organ under a microscope. The total procedure took no longer than 15 min. This preparation will be termed an ‘*in situ*’ cochlea. All reagents were obtained from Sigma‐Aldrich (St Louis, MO, USA) unless stated otherwise.

### Immunohistochemistry

Cochleas were fixed immediately after dissection in 4% paraformaldehyde for 30 min and stored in PBS containing 0.05% sodium azide (NaN_3_), before staining. Primary antibodies against P2X receptors and myosin VIIA were used. These were a P2X2 rabbit polyclonal antibody (APR‐003, Alomone Laboratories, Jerusalem, Israel) and a MYO7A monoclonal antibody, (MYO7A 138‐1, Developmental Studies Hybridoma Bank, Iowa City, IA, USA). Secondary antibodies, Alexa Fluor 488 (A‐11034), Alexa Fluor 568 (A‐11011) and Alexa Fluor 555 (A‐21428), were obtained from ThermoFisher, UK. Primary antibodies were used at 1:500 dilution or 1:250 dilution in the case of MYO7A.

Before staining, cochleas were incubated in blocking solution for 1 h at room temperature. The blocking solution contained: 0.1 mm PBS, 10% goat serum and 0.1% Triton X‐100. After adding primary antibodies cochleas were incubated overnight at 4°C. Subsequently they were washed for 5 min three times with PBS and incubated with secondary antibodies for 3 h at room temperature. Finally, they were again washed three times for 5 min with PBS, and either imaged directly or stored in PBS containing 0.05% NaN_3_ pending imaging.

### Cytoplasmic Ca^2+^ imaging

**Figure 1:**
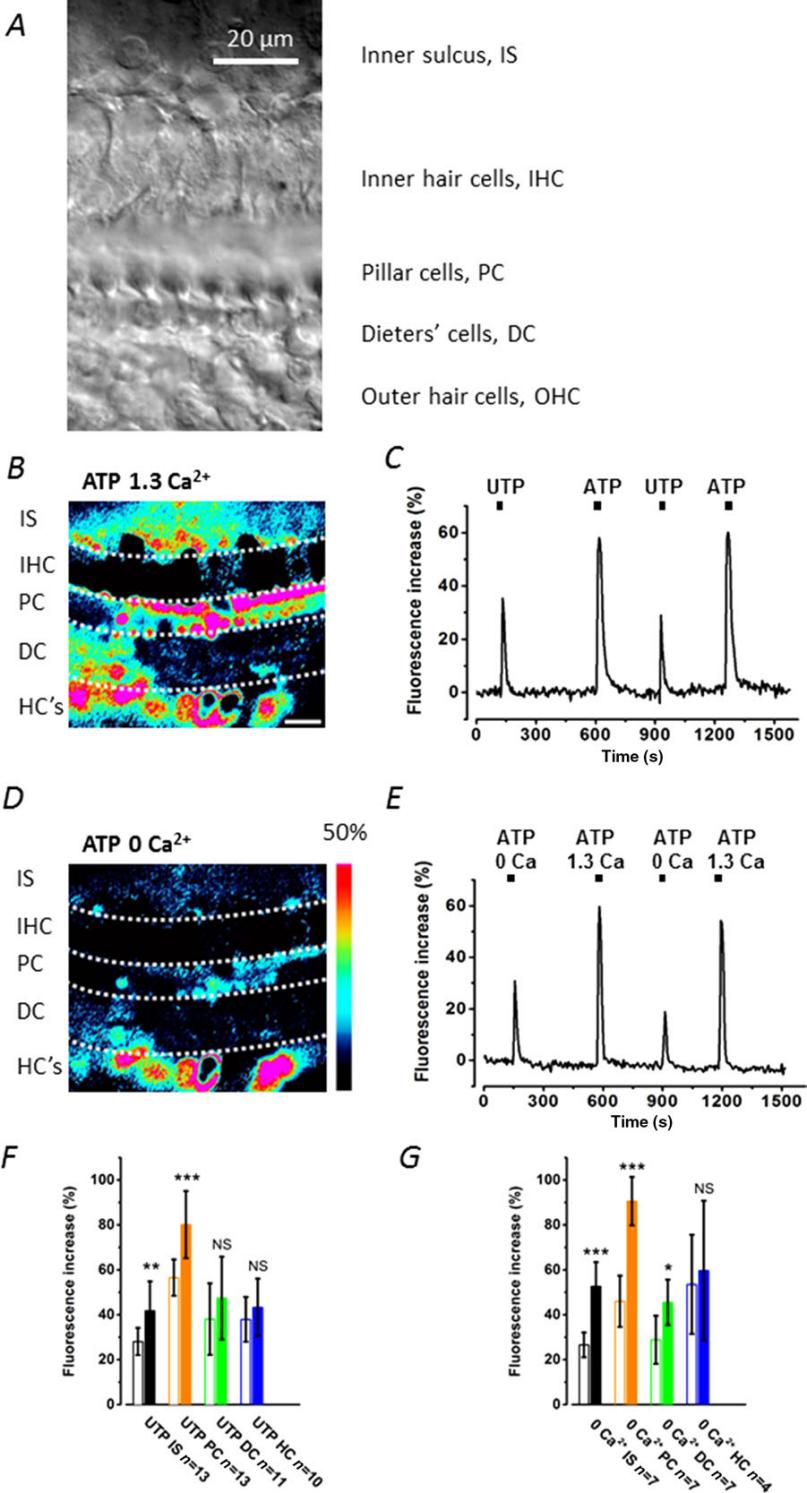
ATP application increases cytoplasmic Ca^2+^ levels in cochlear supporting cells *A*, transmitted light image of the organ of Corti region in the adult *in situ* cochlea. The image shows the different cell types studied. Inner hair cells are distinguished by their large nuclei. The imaging plane, approx. 15 μm below the reticular lamina, shows the region (the arch of Corti) occupied by the inner and outer pillar cells (collectively termed pillar cells, PC) characteristic of the adult cochlea. The Deiters’ cell bodies lie below the OHCs. Scale bar = 20 μm in all images. *B* and *C*, application of 100 μm ATP in normal (*B*, 1.3 mm Ca^2+^) and in extracellular 0 Ca^2+^ (*C*, with 2 mm EGTA) increased cytoplasmic Ca^2+^ levels. The fluorescence changes were measured using 5 × 5 μm ROIs placed over the cells. Signal intensity (%) for the pseudo‐coloured images is indicated by the colour scale bar. Red indicates the highest fluorescence signal. *D*, selective stimulation of P2Y receptors by UTP or by ATP in normal 1.3 mm Ca^2+^, where UTP increased cytoplasmic Ca^2+^ levels less than by ATP stimulation of both P2X and P2Y receptors. Three cochleas for each set of conditions. A single double‐barrel pipette was used to puff‐apply both drugs and solutions. Scale bar as in *B*. *E*, calcium dependence of the ATP responses. Cells were superfused by either 1.3 mm Ca^2+^ or 0 Ca^2+^ (with 2 mm EGTA) while 100 μm ATP was puff‐applied. *F* and *G*, bar graphs comparing the relative fluorescence increase from baseline in inner sulcus (IS), pillar cells (PC), Deiters’ cells (DC) and Hensen's cells (HC) upon stimulation of P2X and P2Y receptors using ATP and UTP. *F*, selective stimulation of P2Y receptors by UTP (open bars) compared to ATP (closed bars) in normal 1.3 mm Ca^2+^. *G*, selective stimulation by 100 μm ATP in 0 Ca^2+^ (with 2 mm EGTA) (open bars) compared to 100 μm ATP in 1.3 mm Ca^2+^ (closed bars). The bath contained 0 Ca^2+^ (with 2 mm EGTA) throughout.

*In situ* cochleae were incubated in extracellular solution with the Ca^2+^ indicator Fluo4‐AM (Invitrogen, Paisley, UK) at a concentration of 20 μm for 45 min at 37°C. Fluo4‐AM was used in all experiments apart from those in which external ATP P2 receptor agonists were applied, in which case cells were loaded with OGB1‐AM with the same protocol. Pluronic acid was present at a concentration of 0.04% (v/v). In preliminary experiments we found that loading into supporting cells with Fluo4‐AM occurred more efficiently than with OGB1‐AM. However, both calcium indicator dyes were used interchangeably in subsequent experiments. In some cases, a ‘nominally zero’ Ca^2+^ (0 Ca^2+^) solution was used in further steps after incubation, obtained by omitting Ca^2+^ from the extracellular solution but compensating for the reduced osmolarity. ‘Nominally zero’ 0 Ca^2+^ was measured to be 60 μm as well as estimated from the specified content of the reagents. In some experiments (e.g. Fig. 1), 2 mm EGTA was included, calculated to reduce free Ca^2+^ to 12 nm.

After incubation, dissected temporal bones were washed in fresh extracellular solution for 10 min before imaging. The temporal bone was attached with cyanoacrylate glue to a 35 mm Petri dish, so that the apical organ of Corti surface faced upwards and could be viewed from above. After loading *in situ* organs of Corti with the Ca^2+^ indicator, the tissue was left without further manipulation in either extracellular solution or nominally 0 Ca^2+^ solution. The tissue could be imaged by confocal microscopy for up to 6 h with no apparent deterioration of the supporting cells. Any such deterioration was identified by visible changes in cell morphology and loss of cytoplasmic fluorescence (Monzack *et al*. 2015). All imaging experiments were conducted at room temperature (21–23°C).

### Image collection and analysis

Cell were imaged using a Zeiss 510 LSM upright microscope using a 40× NA 0.8 water‐immersion objective. The dye was excited at 488 nm, and fluorescence was collected using a 505 nm long pass filter. The pinhole was left fully open to obtain near‐widefield collection. Twelve‐bit images were collected as 560 × 430 pixel frames at a rate of one every 6 s.

For some experiments, to promote cell membrane permeabilization and death, cells were selectively targeted using three pulses of the 720 nm output on the LSM 510 NL0 confocal microscope targeting a 2 × 2 pixel region (corresponding to 3 × 3 μm) using a 100 μs pixel dwell time. This method of permeabilizing cell membranes mimics the approach used previously in the immature organ of Corti (Gale *et al*. 2004).

### Electrophysiological recording

Whole cell tight seal recordings were made using an Axopatch 200B amplifier (Molecular Devices, Sunnyvale, CA, USA). Data were filtered at 10 kHz and sampled using a 1322A interface (Molecular Devices). Data analysis was performed using pClamp 9.0 software. Recording electrodes were made using 1.2 mm outer diameter thin wall capillaries, pulled so that the resistance in the bath was 3–4 MΩ. The intracellular solution contained (mm): 130 KCl, 0.5 EGTA, 2 mgCl_2_, 10 Hepes, pH 7.3. Glucose was used to adjust the osmolarity to 320 mosmol/l.

Dissected temporal bones were stuck upright to glass coverslips and placed in a buffer chamber of a volume of 4 ml with constant perfusion. An upright microscope (Olympus BX50WI) equipped with 10×/0.3w and 60×/0.9w objectives was used for all electrophysiology experiments. Different cells in the organ of Corti could be readily distinguished by their characteristic morphology. The pipettes were guided to identified cells under visual control.

A closed loop perfusion system, with a total volume of approximately 500 ml circulated by peristaltic pumps (Gilson, Middleton, WI, USA) was set up to provide constant perfusion of samples at a rate of 2.5 ml/min. The extracellular solution in the chamber was kept at a temperature of 30°C, using a custom built temperature controller and heating loop.

After obtaining whole cell recording conditions, cells were held at −55 mV. Capacitance, series and membrane resistance were determined from a 20 mV hyperpolarizing step at the start of the experimental runs.

For agonist application, glass pipettes (1.2 mm outer diameter) were pulled to an opening diameter of 1–2 μm. ATP from a concentrated stock was dissolved in extracellular solution to a final concentration of 100 μm and pressure was applied using a pressure injector (PMI‐100, Dagan Corp., Minneapolis, MN, USA) so that the timing could be controlled accurately. In some cases multi‐barrel pipettes were used to avoid unnecessary manipulation and multiple different agonists could be applied to the same section of the organ of Corti. Agonists were applied sequentially allowing at least 300 s between applications to allow the receptors to re‐sensitize.

### Image processing

Fluorescence was measured as the average pixel value in 5 × 5 μm regions of interest (ROIs) placed over identified cell types. Data are presented as fractional increases in fluorescence from baseline. ImageJ and Matlab (Mathworks, Natick, MA, USA) were used for image processing.

Kymographic images (*x–t* plots) were constructed by drawing a curved line along the imaged length of the organ of Corti and measuring the pixel value at every point of this line. Such pixel values were then displayed as ensemble *x–t* scans. Such kymographic images were used to analyse time‐resolved Ca^2+^ wave activity along the Deiters’ cell and IS regions. Images were thresholded using the default automatic threshold function in ImageJ, which is the modified IsoData algorithm implemented in ImageJ ver. 1.41. The binary images established the profile of the Ca^2+^ peaks in the *x–t* plane. They were used to calculate the Ca^2+^ wave travel speed (from the slope), the distance travelled (from the uninterrupted length of the trace) and the average interval between Ca^2+^ waves. To enhance the signal and to show the propagation of the waves, the kymograph series were in some cases constructed from differenced frames separated by six frames (i.e. Δ(n) = image(n) − image(n − 6) for each n), an improvement over differencing adjacent frames.

### Data presentation and statistical analysis

Unless otherwise stated, mean data are shown ± SD. Statistical analysis was carried out such that *P* < 0.05 was considered significant. The Kolmogorov–Smirnov test was used to determine if a normal distribution of data could be discounted. If it could not be excluded that data had a normal distribution, unpaired two‐sample *t* tests were used to compare groups unless indicated otherwise. Unequal variance (Welch correction) was assumed when determining the significance of the difference between groups. Kruskal–Wallis ANOVA was used to compare groups when it could not be assumed that the data had a normal distribution.

## Results

### Extracellular ATP increases cytoplasmic Ca^2+^ in supporting cells of the adult organ of Corti

To determine if purinergic P2 receptors were present in adult mouse cochleas, 100 μm ATP in extracellular solution was puff‐applied to adult organs of Corti. [Ca^2+^]_i_, measured by OGB1 fluorescence, were found to increase in the IS, outer pillar (PC), Deiters’ (DC) and Hensen's (HC) cells (Fig. 1). Responses were also observed in outer hair cells (OHCs) but the imaging focus was not set optimally for such recordings. Since stimulation of both P2Y and P2X receptors could contribute to the observed increase in [Ca^2+^]_i_, 100 μm UTP was puff‐applied in normal extracellular solution to observe the contribution of P2Y receptors to the response (Fig. 1 *D*,*F*).

Cytoplasmic Ca^2+^ increases were observed in the same population of cells when 100 μm ATP was applied in normal (1.3 mm) and in nominally 0 mm extracellular Ca^2+^ solution with 2 mm EGTA added (Fig. 1 *B*,*C*). The increase in fluorescence in 0 Ca^2+^ was significantly lower however. The fractional increase in response to ATP in 0 Ca^2+^ solution compared to normal Ca^2+^ was 0.50 ± 0.11 (*n* = 7 pairs of agonist applications in 3 cochleas) in IS cells, 0.51 ± 0.13 in pillar cells (*n* = 7), 0.63 ± 0.24 in Deiters’ cells (*n* = 7) and 0.90 ± 0.37 in Hensen's cells (*n* = 4) (Fig. 1 *E*,*G*).

Similar results were obtained when 100 μm UTP, a P2Y receptor agonist, was puff‐applied in normal extracellular solution. Application of UTP elicited an increase in [Ca^2+^]_i_. As a fraction of the signal induced by 100 μm ATP in the same cells, the signal induced by 100 μm UTP was found to be at: 0.67 ± 0.15 in IS cells (*n* = 13), 0.70 ± 0.10 in outer pillar cells (*n* = 13), 0.80 ± 0.34 in Deiters’ cells (*n* = 11) and 0.88 ± 0.23 (*n* = 10) in Hensen's cells.

### Exposure to extracellular ATP evokes currents in supporting cells of the adult organ of Corti

To determine if stimulation with ATP could elicit whole cell currents, we recorded from IS cells. These cells replace, or derive from, the cells that form the Kölliker's organ and form the boundary of the cochlear sulcus. IS cells have previously been shown to be extensively coupled by gap junctions in hearing animals (Jagger & Forge, 2006). Coupled IS cells were found to display large variability in input membrane resistance (*R*_m_) between 30 and 660 MΩ (mean 221 MΩ, *n* = 22 cells) and a zero current potential (*V*_0_) between −70 and 9 mV (mean −46.5 ± 19.1 mV). The peak holding current was also found to be variable and at a holding potential of −55 mV lay between 140 and −1300 pA (mean −100 ± 322 pA). The large variability of *R*_m_ and holding current of connected cells was probably due to differences in the extent of coupling between patched cells at the start of the recording. It is also reported that changes in turgor pressure exerted through a patch pipette and osmolarity changes can affect cell coupling in the cochlea (Zhao & Santos‐Sacchi, 1998).

**Figure 2:**
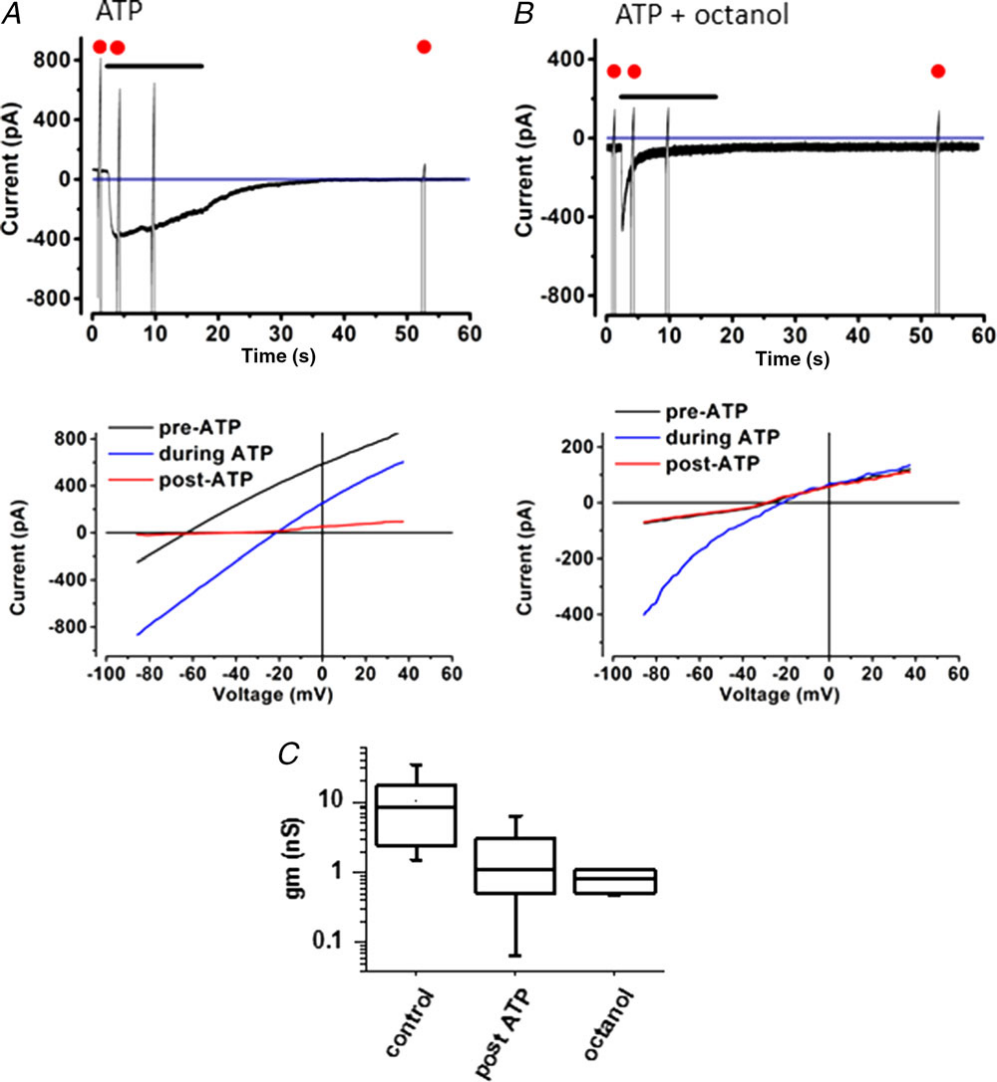
ATP evokes currents in inner sulcus supporting cells *A*, 100 μm ATP evoked inward currents in an IS cell. Voltage command ramps were applied every 60 s. Cells were held at a command potential of −55 mV. Below, *I–V* plot with command ramps (at 0.27 mV/ms) returned towards pre‐ATP application levels. Each of the ramps shown in detail corresponds to a ramp indicated by a red dot shown in the traces. *B*, in the presence of 1 mm 1‐octanol, the response to ATP was shortened with no long‐lasting change in membrane current. *C*, box plot of input slope conductance, g_m_, at *I* = 0 from *I–V* curves. Columns show before ATP (*n* = 22), 60 s after ATP (*n* = 20) and in the presence of 1 mm octanol (*n* = 6). The effect of ATP was to reduce the mean conductance by 11‐fold.

ATP (100 μm) activated a peak inward current in IS cells of 556 ± 316 pA (*n* = 22, range 70–1300 pA). With ATP application, *R*_m_ increased but returned towards pre‐ATP exposure levels with time (Fig. 2 *A*,*B*). Thus, 60 s after the start of a prolonged ATP application *R*_m_ increased to 1.08 ± 0.92 GΩ (*n* = 20 cells, range 33 MΩ to 3.2 GΩ). After a further 2–3 min, the subsequent changes in the current‐voltage curve (*I*–*V*) showed that cells began to return to a partially coupled state (data not shown). These data indicate that ATP uncouples the gap junctional communication and increases the apparent cell input resistance or equivalently reduces the slope conductance (Fig. 2 *C*). The large range in *R*_m_ values after 60s of ATP application shows that adult IS cells exhibit considerable variability in the susceptibility to ATP‐dependent uncoupling in the cochlea *in situ*.

Superfusion of the cochlea with 1 mm octanol, a cell uncoupling agent, increased *R*_m_ approximately fivefold to between 0.21 and 2.10 GΩ (1.30 ± 0.7 GΩ, *n* = 6; see Fig. 2 *B*) although cells still responded to ATP. After uncoupling, the cell input capacitance was in the range 12.7–22 pF, (mean 16.9 ± 3.5 pF) and the zero current potential *V*_0_ was between −27 and −14 mV (mean −21.8 ± 6.2 mV).

Application of 100 μm ATP evoked a peak inward current of between −150 and −1250 pA (mean −472 ± 408 pA, *n* = 6) at a holding potential of −55 mV. These evoked currents showed distinct inward rectification (Fig. 2 *B*).

The responses desensitized with a time constant of approximately 2 s. In the presence of octanol, there was no persistent change in the membrane resistance after ATP application (Fig. 2 *B*). The simplest explanation for the marked resistance change observed in Fig. 2 is that ATP, whilst activating P2X receptors, subsequently produced a Ca^2+^‐dependent closure of gap junctions. Such observations are consistent with results described in the guinea pig cochlea (Lagostena *et al*. 2001).

### Immunohistochemistry of P2 purinergic receptors

**Figure 3:**
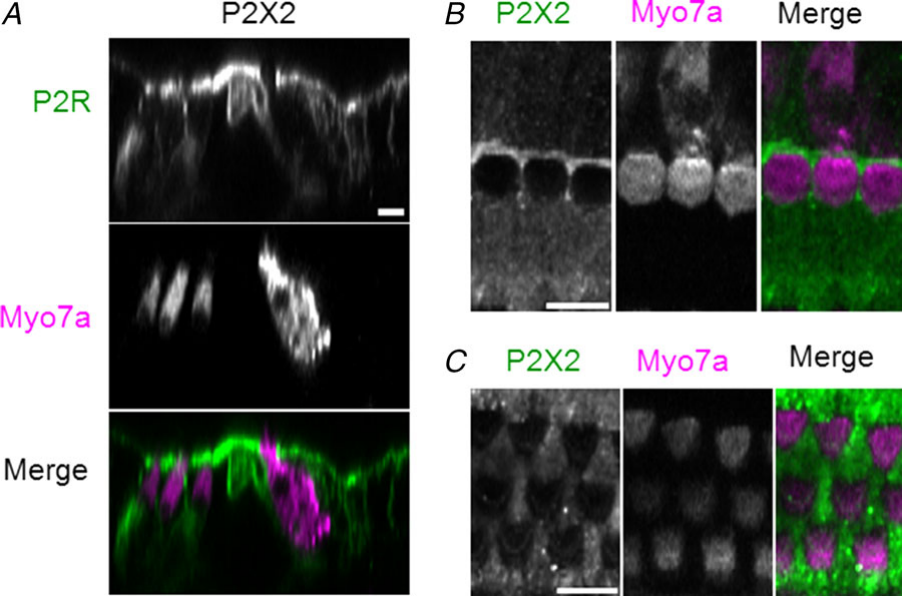
P2X2 receptor expression in the adult organ of Corti *A*, orthogonal projection of P2X2 receptor expression. P2X2 receptor immunofluorescence staining (green), myosin 7a (pink) and merged image. The hair cell cytoplasm is identified using myosin 7a as the label. *B*, surface view of P2X2 receptor immunohistochemistry in the IHC region of the adult mouse organ of Corti. *C*, P2X2 receptor staining in the OHC and Deiters’ cell region. Maximum projection image. Scale bar in all images = 10 μm.

P2X2 receptor expression (Fig. 3 *A*,*B*) was evident in cochlear supporting cells and on the OHC stereocilia (in 98 of 112 cells, 88%; Fig. 3 *C*). A weak signal was also observed in stereocilia of IHCs (19 of 35 cells, 54%). Strong P2X2 receptor staining was observed on the IS cells, border and phalangeal cells, inner and outer pillar cells, Deiters’ cells and Hensen's cells. Between these cells, both types of pillar cells showed the strongest staining at the surface of the epithelium (Fig. 3 *B*). The results of [Ca^2+^]_i_ imaging, electrophysiology and immunohistochemistry are thus compatible with the presence of both P2Y and P2X receptors in the adult mouse organ of Corti.

### Imaging Ca^2+^ spread in the *in situ* cochlea

Two types of spatially propagating calcium transients or ‘Ca ^2+^ waves’ were observed in confocal time‐lapse recordings from the organ of Corti.

**Figure 4:**
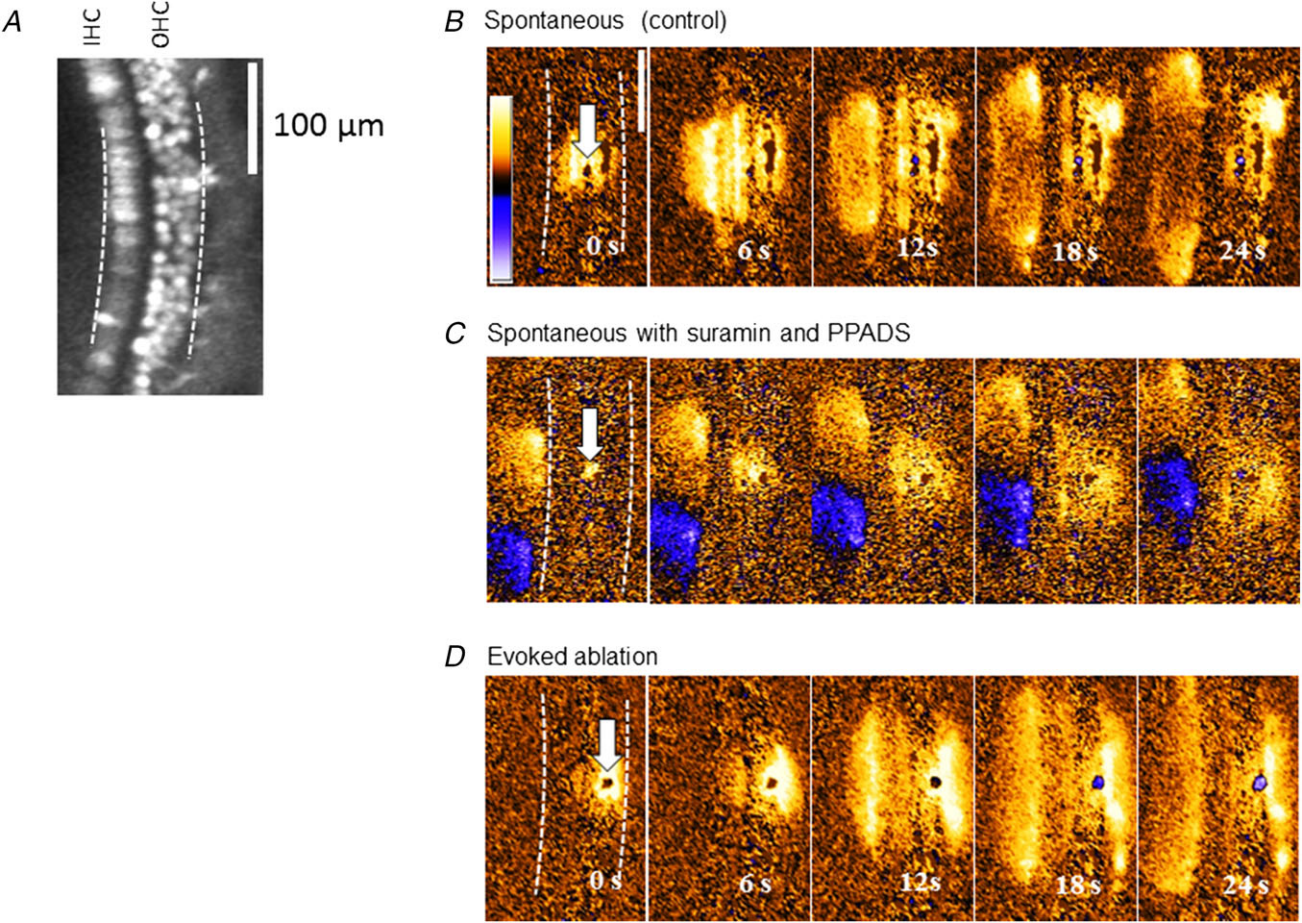
Spontaneous and evoked fast Ca^2+^ waves in the organ of Corti *A*, inner and outer hair cells could be distinguished when loaded with the fluorescent calcium indicator Fluo4‐AM. The number of fluorescent OHCs decreased over intervals exceeding 2 h. Scale bar in all images = 100 μm. *B*, spontaneously occurring waves, propagating from a point indicated by areas of increased fluorescence. *C*, spontaneously occurring waves when P2 receptor antagonists PPADS and suramin were present in the bath, propagating from a point indicated by areas of increased fluorescence. In this example a slow Ca^2+^ wave can be seen propagating in the IS. *D*, Ca^2+^ waves could also be generated following a 720 nm laser pulse focused onto a single cell. The power of the beam was increased progressively until a localized fluorescent spot was observed.

The first type of Ca^2+^ wave was observed whenever there was a sudden loss of fluorescence in an individual OHC. When this happened, a wave spread out from that point. Individual hair cells acting as the origin could be easily distinguished because, using the calcium dye Fluo4‐AM, the hair cells had a stronger fluorescence signal than the surrounding supporting cells (Fig. 4). During prolonged imaging, individual OHCs sometimes spontaneously lost their fluorescence, independently of each other. Once initiated, the loss of fluorescence from an individual OHC was relatively rapid (<6 s) and was typically preceded by an increase from resting fluorescence. In about 5% of cases, the Ca^2+^ signal propagated away from the cell for about 100 μm (or <10 cell diameters) (Fig. 4 *C*). Measurements from two cochleas indicate that the average speed of these Ca^2+^ wavefronts was 6.5 ± 3.4 μm/s (*n* = 10 waves).

These waves differ from those observed after direct injury to hair cells in neonatal cochlear explants (Gale *et al*. 2004) and can be explained by simple ATP diffusion from the point of damage initiating a Ca^2+^ influx. The most economical hypothesis is that such spreading Ca^2+^ signals were initiated by local release of intracellular ATP as a result of breakdown of the OHC plasma membrane. Addition of the P2 receptor antagonists pyridoxalphosphate‐6‐azophenyl‐2′,4′‐disulfonic acid (PPADS) and suramin to the extracellular buffer significantly decreased (*P* < 0.01) the average propagation distance from 104 ± 41 μm (*n* = 10 waves) to 52 ± 19 μm (*n* = 15 waves). There was no significant difference in propagation speed.

To determine whether such waves could be initiated simply by cell membrane breakdown, rather than mechanical disruption, we used a focused Ti‐sapphire laser pulse to permeabilize cells in the Deiters’ cell region (see Methods). This laser permeabilization method initiated Ca^2+^ waves which spread from the targeted site on average for 130 ± 40 μm with an average speed of 7.2 ± 3 μm/s (*n* = 13 waves) from two cochleas.

### Slow Ca^2+^ waves in the organ of Corti

**Figure 5:**
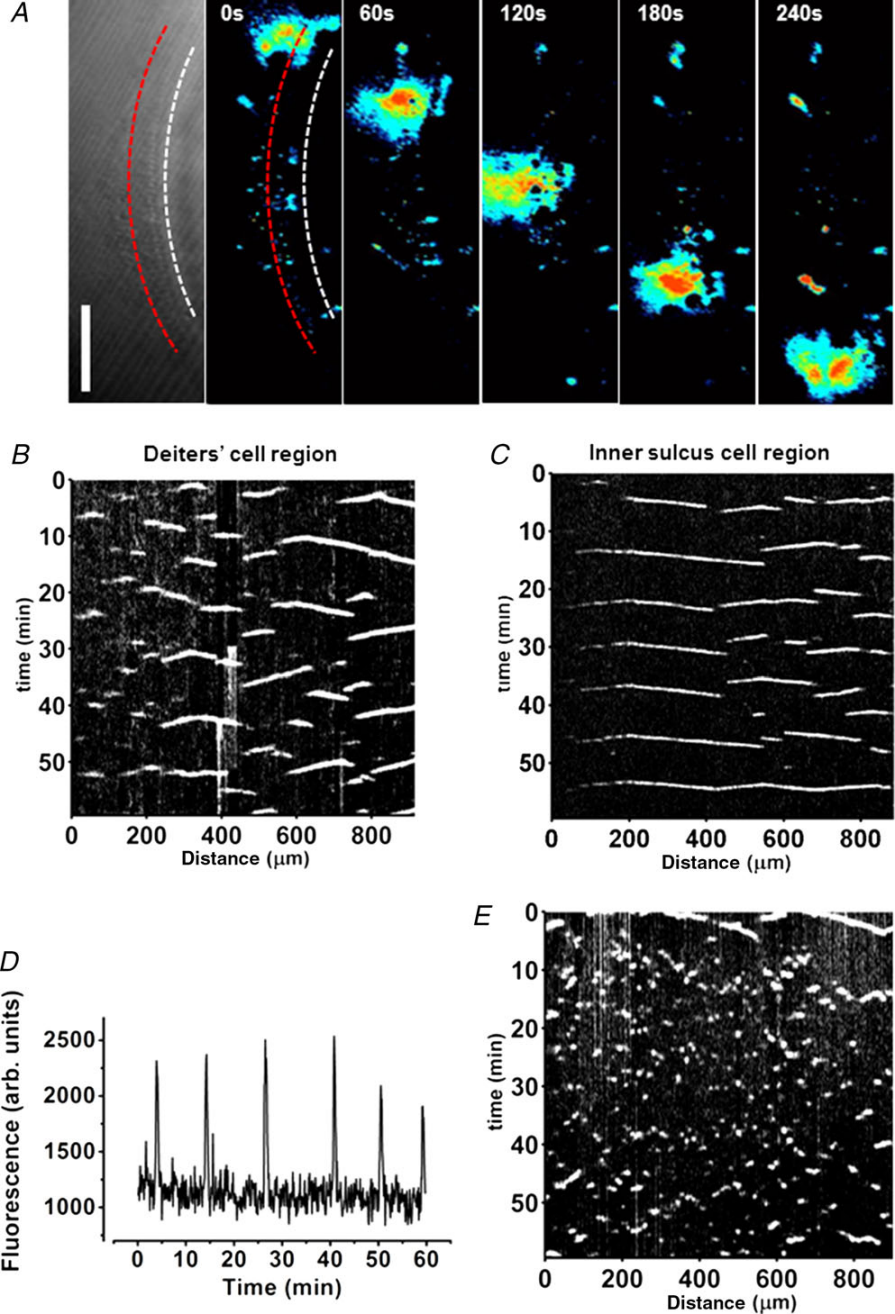
Slow Ca^2+^ waves in the adult organ of Corti *A*, transmitted light image of the *in situ* cochlea (leftmost panel). The tissue was loaded with OGB‐1AM. Spontaneous local Ca^2+^ rises propagated along the tissue. False colour (black, lowest Ca^2+^; red, highest Ca^2+^) background‐subtracted images indicating the change in cytoplasmic Ca^2+^. Fluorescence signal along a line (illustrated here in red) was collected for each frame to construct kymographs (see Methods). The red line depicted here is in the Deiters’ cell region; the white line follows the IS. Scale bar = 100 μm. *B* and *C*, kymographs for the Deiters’ cell region (*B*) and IS cell region (*C*). Black corresponds to the lowest fluorescence, white, the highest fluorescence. The measurement line was drawn along the line of each cell type. *D*, time course of fluorescence changes (arbitrary units) at a single ROI indicating the periodicity of slow Ca^2+^ waves. *E*, addition of the gap junction inhibitor 1‐octanol (1 mm) blocked slow Ca^2+^ wave propagation (reversible after washout, data not shown) resulting in Ca^2+^ signals in individual cells that did not spread.

A different type of Ca^2+^ signalling was also observed in these adult cochleas. Slowly propagating increases in intracellular Ca^2+^ in the organ of Corti (Fig. 5 *A*) were often found which started spontaneously. As will be seen below these waves propagated without diminution of amplitude and at a lower velocity. The characteristics of slow Ca^2+^ waves differed significantly from the damage‐induced and spontaneous waves previously described in the immature organ of Corti (Gale *et al*. 2004; Tritsch *et al*. 2007; Anselmi *et al*. 2008). They also differed from the intercellular Ca^2+^ signals that were induced during cell permeabilization (as in Fig. 4). Initial analysis indicated that this type of Ca^2+^ wave (for which we use the term ‘slow Ca^2+^ waves’) repeated periodically.

Waves were observed to initiate at various positions within the organ of Corti and travelled for variable distances usually in excess of 100 μm. Slow Ca^2+^ waves could be observed separately in the IS region, medial to the IHCs, and in the Deiters’ cell region around the OHCs. Waves terminated after travelling variable distances and the point of termination varied for different waves. The spatiotemporal properties and periodicity of the slow Ca^2+^ waves were analysed using kymograph displays (see Methods), which were constructed for both the Deiters’ cell and the IS regions (Fig. 5 *B*). In such a display, the slope of the *x–t* lines gives the propagated velocity.

Depending upon whether the TM, the extracellular matrix overlying the hair cell bundles, was removed or left in place, the pattern of slow wave propagation differed. Removal of the acellular TM had no detectable effect on the quality of fluorescence imaging. When the TM had been previously removed, slow Ca^2+^ waves were confined to the Deiters’ cell region. In the absence of the TM but in nominally Ca^2+^‐free conditions, slow Ca^2+^ waves propagated in the Deiters’ cell region at a speed of 1.04 ± 0.29 μm/s (*n* = 50 waves, 3 cochleas). This was approximately six times slower than the fast Ca^2+^ waves described earlier. The mean propagation distance was 148 ± 63 μm (*n* = 50, 3 cochleas) with some waves travelling over 300 μm (Fig. 5 *B*). When extracellular Ca^2+^ was increased to 1.3 mm, the slow Ca^2+^ waves were unaffected: the measured propagation speed was then 1.05 ± 0.56 μm/s and distance 130 ± 46 μm (*n* = 29 waves, 3 cochleas). Thus, external Ca^2+^ did not affect the propagation properties of these slow waves and in subsequent experiments normal Ca^2+^ (1.3 mm) was used unless stated otherwise.

When the TM was left in place and attached, slow Ca^2+^ waves were observed not only in the Deiters’ cell region but also in the IS region. In the IS region they travelled at 2.49 ± 0.66 μm/s (*n* = 96, 3 cochleas). This was significantly faster than the wave propagation speed in the Deiters’ cell region under the same conditions (1.44 ± 0.54 μm/s, *n* = 137, 4 cochleas). With the TM attached, the waves propagated on average for 136 ± 70 μm in the IS region whereas in the Deiters’ cell region they propagated for 186 ± 127 μm. Thus, both of the these propagation distances were significantly greater than when the TM was removed (*P* < 0.05).

The presence of the TM, which rests on the surface of the epithelium, thus affected the speed and wave propagation distance. The most probable explanation is that the TM limits the volume of the extracellular space at the surface of the organ of Corti, the reticular lamina. Thus, diffusion of any signalling molecule away from the apical cell surfaces would be limited. The result suggests that restricted diffusion of a local signal could modulate slow wave propagation.

### Determinants of wave propagation

To determine whether gap junctions were involved in propagation of the slow Ca^2+^ waves, 1‐octanol (1 mm) or carbenoxolone (100 μm) were added to the bath to block junctional communication. In these and subsequent experiments the TM was left in place. Exposure to either of the inhibitors blocked propagation of the slow Ca^2+^ waves. In the presence of the inhibitors, spontaneous fluctuations in Ca^2+^ were restricted to individual supporting cells. Without waves there was a somewhat random pattern of Ca^2+^ fluctuations throughout the tissue (Fig. 5 *E*).

**Figure 6:**
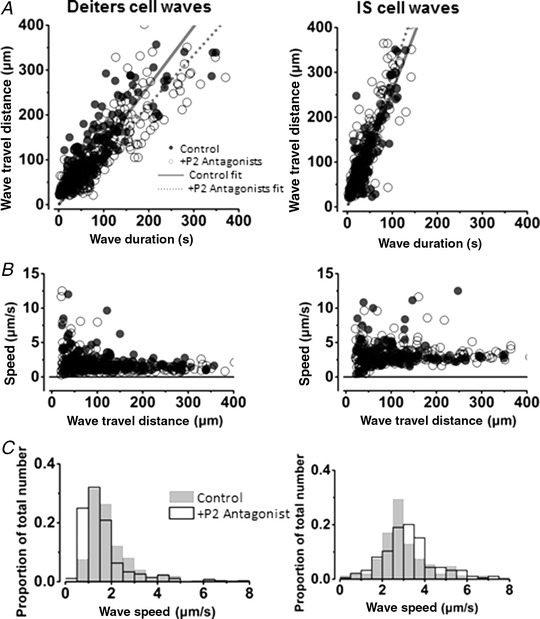
Slow Ca^2+^ waves are not blocked by P2 receptor antagonists *A* and *B*, Ca^2+^ wave propagation distance (*A*) and wave speeds (*B*) for Deiters’ and IS cell regions in 1.3 mm external Ca^2+^. Left‐hand column, Deiters’ cell waves; right‐hand column, IS cell waves. Open circles show wave propagation data in the presence of the P2 receptor antagonists PPADS and suramin. Closed circles show wave data under control conditions with no P2 receptor antagonists. The lines show the fit to the complete data sets: the dotted line shows the fit to the data in the presence of P2 receptor antagonists; the continuous line shows the fit to the control data in the absence of antagonists. *C*, frequency histograms (wave speed) in the presence of (shaded) and absence of (white) P2 antagonists. The slow Ca^2+^ waves in the inner sulcus region propagate faster than slow Ca^2+^ waves in the Deiters’ cell region. Addition of P2 receptor antagonists did not affect wave propagation speed or the distance the wave travelled before extinction in either of the regions.

To determine if slow Ca^2+^ waves depended on ATP receptors, the P2 receptor antagonists PPADS (50 μm) and suramin (150 μm) were added to the bath. With either of these antagonists there was no significant difference in wave travel distance in either the Deiters’ cell or the IS regions (Fig. 6). In addition, there was also no difference in wave propagation distance when apyrase, an enzyme which facilitates cleavage of phosphate from ATP and ADP, was added (40 U/ml final concentration): slow Ca^2+^ waves continued to propagate unaffected (data not shown).

To further implicate gap junctional communication in the appearance of slow Ca^2+^ waves, we found in a limited number of experiments that slow waves were absent in cochleas from connexin 30 knockout mice, whether the TM was present or not. However, fast Ca^2+^ waves were observed in *in situ* cochleas of connexin 30 knockout mice, propagating with the same speed as found in the wild type (data not shown).

### Periodicity of slow Ca^2+^ waves

**Figure 7:**
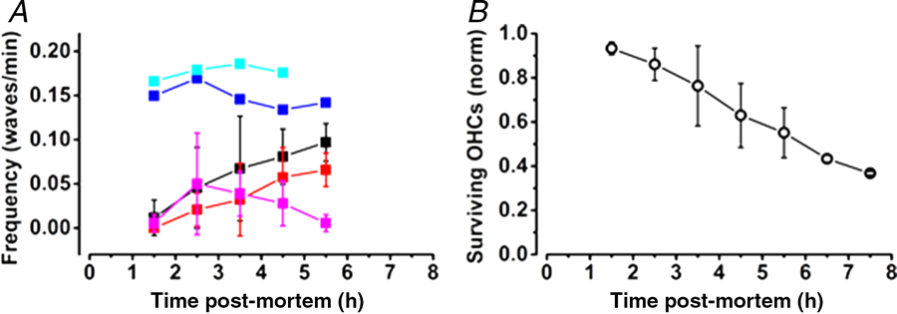
Slow Ca^2+^ wave signalling associated with progressive loss of OHCs *A*, frequency of slow Ca^2+^ waves in the Deiters’ cell region (black) and in the IS regions (red). Measurements made in three cochleas with *t* = 0 signifying removal of the temporal bone. In contrast to the slow wave frequency, fast wave frequency (magenta) decreased after the first hour of observation reaching a peak within the first 2 h after death. When the cochlea was obviously damaged, waves could be observed occurring continuously at a faster rate of approximately one every 7 min. Two examples are shown (cyan and blue). *B*, progressive loss of OHCs as estimated from a count of the fluorescence intensity of the cell profiles over the same period. Thus, 50% of OHC profiles were still present 5 h after death.

Figure 5 *B*–*D* shows that at any one place in either the Deiters’ cell row or the IS cell row there was a clear periodicity to the Ca^2+^ fluctuations, producing a sequence of waves spreading out from the source. To determine the periodicity of the oscillations, the frequency of Ca^2+^ peaks at a fixed spot on the organ of Corti was measured after the start of the experiment. After the first waves were observed at a particular location, the frequency of slow Ca^2+^ peaks reached a maximum within approximately 1 h (Fig. 7 *A*). After onset, the frequency was found to be similar in both the IS and the Deiters’ cell regions reaching 6–7 peaks/h. Thus, for the Deiters’ cell, the mean frequency was 6.3 ± 2.1 waves/h (*n* = 30 measurements) in normal Ca^2+^. For the IS cells the mean frequency was 7.3 ± 1.5 waves/h in normal Ca^2+^ (*n* = 19 measurements). At any given location, none of the different extracellular solutions used, apart from those containing gap junction inhibitors, significantly affected the frequency of these events.

### Onset timing of slow waves

The slow Ca^2+^ waves did not appear immediately after the experiment began, but were obvious within 2–3 h of excision of the temporal bone from the animal. The latency of onset of slow Ca^2^ waves was also affected by removal of the TM. Thus, without a TM slow Ca^2+^ waves were found approximately 120 min after the start of the experiment (in all nine cochleas). When the TM remained in place, slow Ca^2+^ waves appeared later, first being observed approximately after 180 min in both the Deiters’ cell and the IS regions (three cochleas). When the P2 antagonists PPADS (50 μm) and suramin (150 μm) were included in the solutions and the TM was left attached, waves started with a similar delay in both regions.

We quantified the numbers of surviving hair cells in the recordings by using the complete loss of Fluo4 signal as a measure of cell loss. Hair cell numbers began to decline about 2.5 h after death, with only 50% of the hair cells surviving after 5–6 h (Fig. 7 *B*). The decline in OHC survival in the first 1–2 h after the start of observations correlated with the increase in the number of slow Ca^2+^ waves in those preparations.

## Discussion

Using a combination of Ca^2+^ imaging, electrophysiology and immunohistochemistry we have shown that there are propagating Ca^2+^ waves in the adult mouse cochlea. We found that the purinergic receptors present in the IS of the organ of Corti have properties consistent with ATP‐activated ionotropic P2X receptors. The inward rectification and desensitization of these currents are consistent with those of the mouse P2X2 subtype reported in the mouse organ of Corti (Eickhorst *et al*. 2002; Jarlebark *et al*. 2002). However, the ATP‐activated currents of IS cells differ from those reported in Kölliker's organ, a structure of the immature cochlea, where the response is determined by both P2X and P2Y receptors (Tritsch & Bergles, 2010). Since Kölliker's organ is a transitional structure not present in this adult preparation, being replaced by the IS prior to hearing onset (Kamiya *et al*. 2001), we conclude that there is a change in the cell properties on maturation. Consistent with this conclusion, there was no slow second ATP‐activated current component in the adult IS cells characteristic of P2Y receptor activation. ATP signalling in the IS could thus play a different physiological role in the adult and may be reflected in the different Ca^2+^ wave properties. This is consistent with data which suggest that after hearing onset ATP receptors may be involved in reducing damage to the cochlea following high sound levels (Housley *et al*. 2013). In contrast, in pre‐hearing animals, Kölliker's organ's ATP receptors help to depolarize the IHCs and contribute to the correct organization of the auditory system (Wang *et al*. 2015).

**Figure 8:**
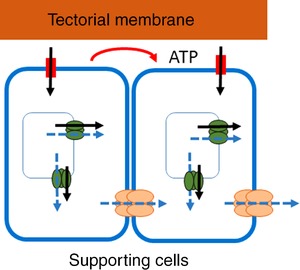
Model comparing fast *vs*. slow Ca^2+^ wave propagation in supporting cells of the adult organ of Corti *Fast waves*: a local extracellular increase of ATP (shown in red) stimulates P2 receptors and increases intracellular Ca^2+^ levels, through a combination of Ca^2+^ influx from the extracellular environment and stimulation of release from intracellular stores. The fast wave propagates primarily through ATP diffusion in the extracellular space (which stimulates P2 receptors, increasing intracellular Ca^2+^ levels). Black arrows indicate the direction of net Ca^2+^ flux during fast waves. *Slow waves*: a trigger event activates Ca^2+^ release from intracellular stores. The signal is transmitted via gap junctions into adjacent cells propagating the Ca^2+^ wave. Dashed blue arrows indicate the direction of net Ca^2+^ flux during slow waves. Wave propagation speed and distribution could be further enhanced by factors present on the TM itself or by modification of the diffusion space around the cells’ apical surface.

We also found that there were two types of propagating Ca^2+^ signals in the adult cochlea. The first was a fast Ca^2+^ wave that depended on extracellular ATP and was initiated at sites of direct or spontaneous damage to OHCs. This wave is very similar to that described in immature cochlear explants (Gale *et al*. 2004). A second type of Ca^2+^ wave propagated about six times slower and was independent of extracellular ATP. It was not initiated immediately by ATP release from damaged or permeabilized OHCs, although it may depend on some signal from those damaged cells. These ‘slow’ Ca^2+^ waves propagated at a constant speed along the organ of Corti in a gap junction‐dependent manner. They were initiated repetitively, often from the same site and travelled unattenuated for hundreds of micrometres through cochlear supporting cells. Figure 8 describes the mechanisms which distinguish these two mechanisms.

In the immature mouse organ of Corti, spontaneous Ca^2+^ waves originating in Kölliker's organ propagate in all directions from the site of initiation (Tritsch *et al*. 2007). The mechanism proposed there is that an initial Ca^2+^ increase in a cell promotes ATP release into the extracellular environment and this ATP activates P2 receptors, promoting an increase in cytoplasmic Ca^2+^ levels in adjacent cells and thus initiating wave propagation. In this case both P2X and P2Y receptors contribute to the subsequent increase in [Ca^2+^]_i_ (Tritsch *et al*. 2007; Tritsch & Bergles, 2010), with an additional activation of Ca^2+^‐dependent channels within Kölliker's organ cells (Wang *et al*. 2015).

The data described herein indicate that the mechanism of slow wave propagation is different. In contrast, these waves are not affected by P2 receptor antagonists and apyrase, indicating that ATP is not critically involved in the production of slow waves. This conclusion is schematized in the model of Fig. 8. Although extracellular Ca^2+^ is known to affect the permeability of hemichannels (Ebihara & Steiner, 1993; Pfahnl & Dahl, 1999), slow wave properties were independent of the extracellular Ca^2+^ concentration, and it thus seems improbable that ATP is released through hemichannels in that case.

### Ca^2+^ waves in the adult organ of Corti

The results show that Ca^2+^ waves propagating between cells can still be spontaneously activated in the *in situ* organ of Corti and may well be spontaneously active in the organ of Corti well after hearing onset (Sung *et al*. 2003; Lahne & Gale, 2008). In the adult organ of Corti, the presence of the TM affects the propagation speed and hence indicates that a diffusible factor may be involved which is as yet unidentified.

Waves with properties similar to those of the observed slow Ca^2+^ waves have not been observed previously in the adult organ of Corti. Ca^2+^ wave propagation has been observed in a related preparation of the adult gerbil explant under conditions where the cochlea was acoustically overstimulated, although such waves had very similar characteristics of speed and propagation distance to the fast waves reported here (Chan & Rouse, 2016). Similarities do exist with waves found in multiple other tissues, where such waves are thought to play an important role in synchronizing and regulating cell physiology. As demonstrated in the case of ATP‐dependent Ca^2+^ waves in immature cochlear supporting cells, transient increases in cytoplasmic Ca^2+^ in cells could evoke a physiological response including kinase activation and potential downstream changes in gene expression patterns (Lahne & Gale, 2008; Ortolano *et al*. 2008).

Similar calcium waves have been observed in many cell types including astrocytes (Cornell‐Bell *et al*. 1990), epithelial (Nihei *et al*. 2003) and endothelial cells (Vandamme *et al*. 2004), reviewed in (Leybaert & Sanderson, 2012). Intercellular Ca^2+^ waves with similar properties have also been reported during development in the epibolizing blastoderm (Webb & Miller, 2006; Kurth‐Nelson *et al*. 2009), during morphogenesis in the zebrafish embryo (Creton *et al*. 1998) and during neural induction in *Xenopus* (Leclerc *et al*. 2000). In this last system, wave inhibition was observed to decrease gene expression associated with neural development.

The periodic and active nature of the observed ‘slow’ Ca^2+^ waves may indeed implicate them in gene regulation. Periodic oscillations of [Ca^2+^]_i_ are known to regulate gene expression (Dolmetsch *et al*. 1998). It is thought that different transcription factors and transcription factor regulators are sensitive to different frequencies of Ca^2+^ oscillations (reviewed by Smedler & Uhlen, 2014). The periodicity of Ca^2+^ oscillations described here falls in the range of oscillations that are recognized by such transcription factors as MAPK and NF‐κB. The frequency of oscillations could be higher *in vivo* because the present experiments were done at room temperature of (21–23°C) and temperature is known to affect cytoplasmic Ca^2+^ signalling. For example, in other tissues Ca^2+^ wave speed has a temperature dependence, Q10, which has been estimated to be between 2 and 3 (Cosens *et al*. 1976; Jaffe, 2002).

Intercellular Ca^2+^ waves have been linked to the regulation of neuronal division in the ventricular zone of the neocortex (Weissman *et al*. 2004). In the mammalian cochlea, the cells in the organ of Corti go through terminal cell mitoses during early development (Ruben, 1967; Chen & Segil, 1999) and hair cell regeneration is not known to occur in the mammalian cochlea although regeneration is a feature of other vertebrate hearing organs (Groves, 2010). Nevertheless, extracellular ATP‐dependent Ca^2+^ waves and associated intracellular Ca^2+^ oscillations in cochlear supporting cells have been shown to lead to the activation of Erk signalling in surrounding supporting cells and the control of hair cell death (Lahne & Gale, 2008). Here, slow Ca^2+^ waves are also associated with OHC loss (Fig. 7).

Our observations reveal sustained periodic increases in intracellular Ca^2+^ levels which propagate among cochlear supporting cells. The role and physiological effects of these waves are not yet clear but their nature may suggest a role in gene regulation. Thus, fully elucidating the nature and consequences of slow Ca^2+^ waves could increase our knowledge of the processes leading to hair cell loss and give insights into possible avenues which could lead to hair cell regeneration.

## Additional Information

### Competing information

None declared.

### Author contributions

Conception and design of the experiments: P.S., J.E.G. and J.F.A.; collection of the data: P.S.; analysis and interpretation of the data: P.S., J.E.G and J.F.A.; drafting the article or revising it critically for important intellectual content: P.S., J.E.G. and J.F.A. All authors approved the final version of the manuscript. All experiments were carried out at the UCL Ear Institute.

### Funding

This work was supported grants from the Medical Research Council Laboratory of Molecular and Cell Biology studentship programme (Ref MC_U12266B) to P.S. and from the Wellcome Trust to J.F.A (Ref 093084).
